# Reconceptualization of eating addiction and obesity as displacement behavior and a possible treatment

**DOI:** 10.1007/s40519-022-01427-1

**Published:** 2022-06-22

**Authors:** Robert Pretlow, Suzette Glasner

**Affiliations:** 1 eHealth International Inc., 2800 Elliott Ave. #1430, Seattle, WA USA; 2 UCLA Integrated Substance Abuse Programs, David Geffen School of Medicine, Los Angeles, CA USA

**Keywords:** Displacement, Mechanism, Behavior, Eating, Addiction, Obesity

## Abstract

**Purpose:**

Displacement behavior is a biobehavioral mechanism that allows an animal to deal with situations that cannot readily be faced nor avoided, or that are thwarting. It may explain compulsive overeating (eating addiction). Resembling addiction, displacement behavior is irrepressible behavior that is contextually inappropriate, e.g., sleeping or feeding when threatened by a predator, or binge eating in response to a work altercation. It is thought to be due to rechanneling of overflow brain energy to another drive (e.g., feeding drive) when two drives, e.g., fight or flight, equally oppose each other. Moving the opposing drives out of equilibrium, by resolving the person’s underlying problems/stressful situations, theoretically should mitigate the displacement mechanism and addictive overeating.

**Methods:**

We developed a mobile phone intervention targeting addictive overeating, including a displacement mechanism component. A displacement use subgroup (*N* = 37) ages 14–18 with obesity (mean BMI = 38.1) identified life situations they could neither face nor avoid, or that were thwarting them, and developed action plans to address each situation. Feasibility and acceptability were evaluated.

**Results:**

Participants found the displacement component to be understandable and user-friendly. The majority (26/37–70%) used the core “Dread List” feature to input 90 individual dreaded/problem situations fueling displacement-based overeating, coupled with action plans to address each problem. Dread items related to school accounted for nearly one-half (46%: 41/90) of all dread situations reported by participants.

**Conclusion:**

The displacement mechanism may be a useful basis for treatment of eating addiction and obesity and may provide individuals with hope that they can curb their addiction without relying on willpower to not overeat. A randomized trial evaluating the displacement intervention is planned.

**Level of evidence:**

Level V: Opinions of respected authorities, based on descriptive studies, narrative reviews, clinical experience, or reports of expert committees.

**Registration:**

The study was reported according to the Consolidated Standards of Reporting Trials (CONSORT) statement and was registered with ClinicalTrials.gov (NCT03500835) April 18, 2018.

## Introduction

Treatments for obesity, which commonly focus on diet and exercise, have yielded mixed results, and are typically implemented in specialty care settings, limiting generalizability of study findings. Novel intervention strategies for obesity are urgently needed, and the addiction treatment literature may offer some of the most relevant potential models to inform their development. The current paper is a follow-up to our previous publication in this journal [1], in which we conceptualized eating addiction as having sensory (e.g., taste, texture) and motor (e.g., crunchy, chewy) components, with a specific treatment for each component. In the current paper, conceptualization of eating addiction is further evolved as displacement behavior, with a suggested treatment.

Leaders in addiction science concur that addiction and obesity both reflect the consequences of ingestive behavior gone awry. The core similarities between these conditions can be summarized as follows. First, in terms of clinical diagnostic features, both addiction and obesity result from repetitive foraging and ingestion behaviors that intensify and persist despite negative and (at times) devastating health and other life consequences. Likewise, despite often repeated attempts to reduce or quit using addictive substances, relapse is common in the addiction recovery process, just as those with obesity who attempt to regulate their food intake through dieting frequently relapse and return to their elevated body weight. Second, only a subset of individuals who are exposed to substances with addictive potential develop addictive behaviors, just as not all people who are exposed to foods and diet patterns that pose difficulties with weight control become obese. Nevertheless, a central barrier to the success of treatment for obesity that is distinct from drug addiction is the fact that food consumption is essential for survival; thus, abstinence is not a feasible or appropriate treatment goal. Accordingly, understanding and targeting the behavioral and psychological precursors to compulsive eating behaviors is essential as a means of facilitating control over food intake to mitigate obesity.

Stress is a precursor that is common both to compulsive eating behavior and alcohol/drug use. Stress in childhood has been shown to predict weight problems during early adolescence and young adulthood [2], with parallel findings in the addiction literature [3]. One putative explanation for the association of stress and associated life problems with addictive behaviors is offered by displacement theory.

### Displacement behavior

Displacement behavior is an innate, biobehavioral mechanism in the brains of all animals, from fruit flies [4] to humans [5]. It functions as a response to situations that the animal cannot readily face, yet cannot avoid—situations involving uncertainty, confusion, conflict, or a feeling of being trapped, threatened, or frustrated—herein defined as “stressful situations.” Displacement behavior is a normal behavior or drive (such as licking or grooming) that occurs out of context (e.g., when threatened). Although it is adaptive, if displacement behavior is excessively practiced, it may become destructive; for example, socially isolated, stressed dogs may lick their paws raw [6]. Eating can function as a displacement activity and also potentially lead to maladaptive outcomes. For instance, sheep threatened by a predator will graze despite the danger [7]. Similarly, both male turkeys and cocks, when fighting, will suddenly stop and eat, if food is available, even though they are not hungry, and subsequently resume fighting [8].

Displacement behavior bears a striking resemblance to addictive behavior. Like addictive behavior, displacement behavior is (1) irrepressible or out of control, and (2) out of context (i.e., not an appropriate response in the various sets of conditions in which it occurs), e.g., an animal that sleeps or feeds when threatened by a predator or an individual who drinks to intoxication or binge eats after encountering a stressor in the workplace. Thus, we hypothesize that an underlying mechanism in the genesis and maintenance of addictive behavior is the brain’s displacement behavior mechanism going rogue and developing a life of its own.

Displacement behavior is thought to result from the rechanneling of overflow brain energy to another drive (e.g., feeding drive) when two drives, e.g., fight vs. escape, equally oppose each other [8]. Moving such opposing drives out of equilibrium, by (1) helping the individual to identify the problem(s) or stressor(s) that form the basis of the opposing drives, thereby fueling overflow brain energy (i.e., displacement sources) and (2) assisting the person in forming strategies to either avoid or effectively resolve these problems/stressors, could form a behavioral intervention approach for targeting the displacement mechanism believed to be underlying addictive behaviors, including eating addiction and obesity.

The displacement mechanism is triggered by sensory cues [7]. Initially, a specific sensory cue (e.g., food taste) suggests to the brain that the behavior or drive (e.g., feeding) associated with the cue might be used as a displacement behavior to deal with problems/stressors/thwarting and overflow brain energy. The brain then appears to lock onto the respective drive, and henceforth similar cues trigger the displacement mechanism to activate that drive behavior (eating) in stressful situations. Triggering of that drive by the sensory cues may be self-reinforcing when problems/stressors/thwarting are present, to a point that the displacement behavior may become excessive and destructive (e.g., overeating/obesity).

We propose that the displacement mechanism phenomenon might be expressed as an equation, as depicted in Table 1, using the examples of skin picking and overeating behaviors. Note: it is not muscle energy that is thought to be expended by the displacement mechanism, but rather brain energy or mental energy. Displacement behavior involves intense focus on the respective medium and cues (e.g., rough skin, tempting food), which ostensibly is how overflow mental energy is expended. Rechanneling diverts the focus to a nondestructive medium (e.g., squeezed fist). Dealing with the displacement sources will diminish the focus on the destructive medium and cues. Variables of the displacement equation might be quantified by validated questionnaires.

**Table 1:** Proposed equation expression of the displacement mechanism ${\text{D}}\,{ = }\,{\text{S}}\,{ + }\,{\text{C}}\,{ + }\,{\text{M}} - {\text{R}}$

Variable	Name	Explanation
D	Displacement	Displacement severity (e.g., degree of skin picking, overeating)
S	Source	Source of the displacement (e.g., stressful life situations and thwarting): level, quantity, acute vs. chronic
C	Cue	Cue triggering the displacement: (e.g., cuticle raggedness and food sensations level): cue sensitivity, cue availability, and cue quality
M	Medium	Displacement medium (e.g., ragged cuticles and tempting foods): quantity, quality, and availability
R	Rechanneling	Rechanneling the displacement (e.g., deep breathing, squeezing hands, and hobbies): quantity, quality, and availability

In the present study, we investigated the feasibility and acceptability of therapeutic techniques based on the displacement theory through the inclusion of a displacement-intervention component to an mHealth intervention that was part of a larger ongoing randomized clinical trial (RCT) targeting overeating behaviors among youth. While a series of theoretical contributions on displacement activity, primarily in animals, flourished from approximately 1950–1970, there has been little added to the literature thereafter. Here, we seek to examine the application of displacement theory as a novel treatment for eating addiction and obesity. The current study used qualitative data collected from a subgroup enrolled in the RCT to determine if participants find the displacement feature of the smartphone application to be helpful in changing eating behaviors and to identify the problematic life situations that participants believed may putatively fuel displacement-based overeating behavior.

## Methods

### Participants

As part of a larger 6-month pragmatic trial (*N* = 180) of an addiction-based smartphone app weight-loss intervention in obese young people who were recruited from multidisciplinary weight-management clinics at four sites, the displacement mechanism intervention was introduced to a subgroup (*N* = 37) of participants, 53% recruited from Children’s Hospital Los Angeles (CHLA), 7% from Cedars Sinai, 18% from Harbor UCLA, and 18% from UCLA. The subgroup study took place between October 2019 and December 2020, during which Los Angeles was experiencing a high number of Covid cases, with disruption to school, work, and social life. Study procedures were approved by the CHLA Institutional Review Board. Written informed consent was obtained from the participants (and one parent or guardian if the participant were a minor).

To participate in the study, participants needed to be 14–18 years old, have a BMI ≥ 85th percentile for age and gender, and the ability to read and understand English. Participants were excluded if they were already participating in another weight-loss intervention, had a blood pressure > 99th percentile for age, gender, and height, or if they had a known poorly controlled psychiatric illness or developmental delay per their primary care physician.

Participants were an average of 17.1 years old (SD = 1.9, range 14–18) and had a mean BMI of 38.1 (SD = 9.4). The majority of participants were Latinx (65.5%), female (82%), publicly insured (73.88%), and had an annual household income of less than $50,000 (71%).

### Procedure

As part of the larger trial, all participants had access to a smartphone app that was designed to help them abstain from problem foods, eliminate snacking between meals, and reduce excessive amounts at meals. Those in the present study were given access to an added feature of the app targeting problematic life situations that putatively fuel displacement-based overeating behavior. Using this feature, termed the “Dread List,” participants were provided with an explanation of the displacement theory and its relationship to overeating behavior. As a means of reducing their displacement behavior, they could enter: (1) life situations that they dreaded or could not readily face, yet could not avoid, or situations with which they were frustrated; and (2) “action plans,” or problem-solving approaches to each situation. The mantra, “Face it… don’t displace it,” was included in the app to inspire participants to use the Dread List feature. A point-accrual system allowed participants to earn points as they completed tasks, which were associated with a dollar amount; for each Dread List item that they entered, they earned 15 cents compensation. Assessment of feasibility and acceptability of the Dread List app component was based on use of this feature.

### Data analysis

The open-ended responses to the Dread List were coded inductively with quantitative descriptive measures analyzed by Fishers exact test.

## Results

The majority (26/37–70%) of participants in the displacement-intervention subgroup used the core Dread List feature to input a total of 90 (*M* = 3.5, SD = 2.3) individual problems/stressors fueling displacement-based overeating, coupled with 161 (*M* = 6.2, SD = 4.1) corresponding problem-solving/action plans.

Table 2 shows examples of the eight dread list categories identified from the coded open-ended text responses with corresponding example action plans.

**Table 2:** Thematic dread list example situations and action plans

Dreaded situation	Action plans
School: big assignments in school (mainly Math and English)	Plan and take small steps at a time to not get overwhelmed. Prioritize assignments
Mental: I do not like not having motivation to do things or feeling useless or without purpose	I am going to find something I am passionate about and make a schedule every day to do something productive and do something fun
Weight: breaking the snacking rule	Possibly find new hobbies to keep me entertained or distracted enough
Family: my dad was hospitalized and I felt extremely lonely	Spend more time with my dad
Activities: being home all day	Draw. Cook. Play video games. Facetime your bestie
Health: diabetes—could get it	Eat healthier food
Friends: friends that will hang out with me	Keep myself busy and cultivate relationships
Financial: studio tuition	Create payment plan

Table 3 presents the frequency of the eight dread list categories by whether the dread item was related to Covid. Of the 26 participants using the Dread List feature, 20 (77%) reported one or more dread items related to school, with the school category accounting for nearly one-half (46%: 41/90) of all dread situations. The school-related dread items detailed concerns about forthcoming tests, the amount of homework assignments, and the difficulty of the schoolwork. Dread situations categorized as “Mental” were reported next frequently (16%: 14/90) and included feeling powerless, low energy, sleeping difficulties, feeling trapped indoors and slipping back into depression. Fourteen percent (13/90) of dread situations related to weight issues and included not breaking the snacking rule, weight fluctuating and feeling like they were getting fatter. Just over one-quarter (28%) of dread situations contained a Covid-related element, e.g., concerns about school tests which were going to be administered on-line (due to Covid) or being unable to receive face-to-face assistance from teachers due to teaching being conducted remotely. A Fishers exact test was used to determine whether there was a significant association between the dread list categories and whether the dread was related to Covid. There was a statistically significant association between dread list categories and Covid (*P* = 0.002) with higher proportions of Covid-related items in the activities, friends and school categories.

**Table 3:** Frequency of dreaded situation category and whether related to Covid

Dreaded situation category	*N* of participants*	Covid-related		Total
		No	Yes	
School	21	30 (71%)	12 (29%)	42 (47%)
Mental	6	12 (86%)	2 (14%)	14 (16%)
Weight	5	12 (100%)	0 (0%)	12 (13%)
Family	7	5 (71%)	2 (29%)	7 (8%)
Activities	5	1 (14%)	6 (86%)	7 (8%)
Health	4	3 (75%)	1 (25%)	4 (4%)
Friends	3	1 (33%)	2 (67%)	3 (3%)
Financial	1	1 (100%)	0 (0%)	1 (1%)0
Total	26	65 (72%)	25 (28%)	90 (100%)

*N of participants reporting a dreaded situation in each category

Anecdotally, it was observed that cue sensitivity decreased if displacement sources were addressed by action plans. One example involved a 20-year-old female, 5′ 9′, 187 lbs. Before leaving work at the end of the day, she would identify difficult situations in her life and create an action plan to deal with each one. After doing so, she was surprised that when driving home past McDonald’s and Taco Bell, she no longer was tempted to stop and binge.

## Discussion

Nearly three-quarters of participants evidenced engagement with an mHealth intervention component that presented the displacement theory and provided an opportunity to address overeating as a displacement behavior. Results suggest that presenting the displacement mechanism theory via an mHealth platform is feasible and that a psychosocial intervention component targeting the stressors or problems that may fuel eating as a displacement behavior is acceptable to young people who suffer from obesity.

Dread list results were revealing. School was identified as a major source of stress (that is consistent with other research) that could lead to overeating. A notable finding was that Covid added to this stress, particularly for school activities. The endeavor of trying to lose weight was a stressor for some, which might lead to a vicious cycle of displacement overeating.

Habit-reversal therapy is used to treat maladies like skin picking, yet it seeks only behavior change and does not treat the underlying cause [9]. We posit that the basis of destructive habits is the displacement mechanism and, therefore, warrants further investigation regarding its utility as an important component of interventions targeting addictive behaviors.

### Displacement quandary

A perplexing aspect of the displacement mechanism is why it becomes excessive and destructive in some individuals—that is, why do some people abuse drugs/alcohol and food, yet others do not? We speculate that those individuals may lack basic coping mechanisms and are unable to face, avoid, adapt to, or solve their underlying problems. We further speculate that for such persons, the destructive displacement behavior may become their sole coping avenue, may be self-reinforcing, and may reach a “point of no return”.

### Rechanneling the displacement

Theoretically, the displacement mechanism functions by rechanneling overflow mental energy to another behavior, typically whatever behavior is most readily available at the time or is most commonly used in the animal’s repertoire, e.g., grooming and feeding [7]. If the rechanneled behavior becomes destructive, it is possible for the individual to consciously rechannel the overflow mental energy to a nondestructive behavior. Examples are rechanneling to breathing behavior (by taking slow, deep breaths), rechanneling to squeezing the hands, and rechanneling to hobbies [5].

We hypothesize that the displacement mechanism is a driving force behind eating addiction and obesity. The addiction field, on the other hand, as well as the obesity field, has emphasized the role of the reward mechanism in both alcohol/drug addiction and obesity [10]. We acknowledge that the reward mechanism is a central component underlying addictive eating behavior, but we posit that rewards (e.g., pleasurable food sensations and celebrations) rather act as cues to trigger the displacement mechanism, leading an individual to lose control over eating, once started. Activation of the (irrepressible) displacement mechanism may explain why individuals feel compelled to overeat or binge in the face of a rewarding cue yet feel substantial regret afterward. Regret felt by individuals after a binge episode could also be, at least in part, due to social pressure, diet culture and potential effects on body weight. Regret would not occur if it were simply a matter of reward. Contrite displacement behavior would thus seem responsible for regret. Reward and displacement are, therefore, interconnected, and theoretically one would not occur without the other. Nevertheless, per the displacement equation, difficult or thwarting life situations may trigger the displacement mechanism with minimal rewarding cues, leading an individual to overeat whatever food is available in the moment [1]. Likewise, highly pleasurable foods may trigger displacement overeating in the absence of acute stress and in the presence of only chronic background stress.

As underlying mechanisms of addiction, aberrant displacement and reward mechanisms could ideally be addressed in an integrated treatment approach. The displacement mechanism may be treated by (1) identifying life situations the person cannot readily face yet cannot avoid, and (2) implementing action plans to effectively address each situation. The reward mechanism may be treated by teaching the individual to identify and avoid reward cues, which in turn would mitigate triggering of the displacement mechanism.

In our previous study [1], we tested a treatment for hypothesized sensory and motor components of eating addiction, with the motor component felt to be predominant. The sensory component consists of the taste, texture, and temperature of food and was treated with staged food withdrawal/abstinence, whereas the motor component consists of the actions of eating such as crunching, chewing, sucking, and swallowing and was treated with methods used for body-focused repetitive behaviors. We now realize that the motor component is in fact the displacement mechanism, and the sensory component is the cue-reward mechanism that triggers the displacement mechanism.

In the present study, we developed and evaluated the feasibility of a technology-assisted intervention component targeting the displacement mechanism in young people with obesity; however, this intervention does not address the cue-reward mechanism. Thus, a future iteration of this approach would necessitate developing one or more features to help individuals identify reward-based overeating cues and decrease their sensitivity to them. The current intervention also does not use rechanneling of a destructive displacement behavior to a nondestructive behavior. To enrich our displacement mechanism intervention, we are adding features that address the displacement cues and the rechanneling of destructive displacement behavior and are preparing to investigate the preliminary efficacy of an mHealth application, based solely on the displacement mechanism, in a pilot randomized clinical trial.

### Strength and limits

The strength of the current study lies in the remarkable ability of the majority of these young people to identify difficult situations and frustrations in their lives and create thoughtful and compelling action plans to deal with each one.

Several limitations of the study warrant comment. First, given the preliminary nature of the displacement-intervention strategy, coupled with the absence of a control condition, we did not examine its impact on clinical outcomes, such as weight loss, but report only on feasibility of the displacement method. Second, we were not able to directly assess participants’ understanding of the displacement theory or whether they effectively implemented any of the “action plans” they developed. Thus, the impact of the Dread List exercises on participants’ eating behaviors, problem-solving abilities, and functional outcomes remains unknown. Third, apart from participants’ use of the Dread List, we do not have data to indicate how helpful they found this component. Lastly, participants’ motivation for engaging with the displacement component of the app may have been largely extrinsically driven (i.e., monetary reward). In our pilot trial we plan to target youth via a screening process prior to enrollment, who are more intrinsically motivated to change their eating behaviors, as well as collect the data outlined above to address the limitations of the current study.

## Conclusions

Reconceptualization of eating addiction and obesity as displacement behavior may be warranted. The proposed displacement intervention of problem solving and rechanneling destructive displacement behavior appears to be a feasible approach to the treatment of eating addiction and obesity in young people. One advantage of displacement intervention over conventional diet, exercise interventions, as well as abstinence-based addiction interventions, is that direct substance/food restriction is not required. If the displacement mechanism accounts for overeating, then targeting this mechanism in treatment should facilitate significant reductions in overeating without necessitating willpower to eat less.

## Declarations

### What is already known on this subject?

A succession of observational discussions on displacement activity, primarily in animals, appeared in the literature in the 1950s and 1960s.  Nevertheless, displacement theory was regarded as more of a curiosity than a useful concept and was never applied to human psychology, particularly treatment of clinical disorders. Nothing really new has appeared in the literature after about 1970.

### What does this study add?

We applied displacement theory as a novel intervention for addiction, specifically eating addiction and obesity. We found that a majority of obese young people remarkably were able to identify their difficult life situations underlying displacement behavior and create compelling action plans to deal with each one. These results support subsequent development of an intervention for eating addiction and obesity based on displacement theory and implemented as a smartphone app with a clinical trial.
