# Correction: Reconceptualization of eating addiction and obesity as displacement behavior and a possible treatment

**DOI:** 10.1007/s40519-024-01644-w

**Published:** 2024-02-13

**Authors:** Robert Pretlow, Suzette Glasner

**Affiliations:** 1 eHealth International Inc., 2800 Elliott Ave. #1430, Seattle, WA USA; 2 UCLA Integrated Substance Abuse Programs, David Geffen School of Medicine, Los Angeles, CA USA

**Correction: Eating and Weight Disorders - Studies on Anorexia, Bulimia and Obesity (2022) 27:2897–2903** 10.1007/s40519-022-01427-1

In this article [1], the Keywords was not displayed correctly. The correct Keywords are listed below.

**Keywords**

Eating addiction

Displacement mechanism

Displacement behavior

Displacement activity

Obesity
